# Impact of Larval Food Source on the Stability of the *Bactrocera dorsalis* Microbiome

**DOI:** 10.1007/s00248-024-02352-9

**Published:** 2024-02-26

**Authors:** Vivek Kempraj, Jean Auth, Dong H. Cha, Charles J. Mason

**Affiliations:** 1 USDA-ARS, Tropical Crop and Commodity Protection Research Unit, Daniel K Inouye US Pacific Basin Agriculture Research Center, Hilo, HI 96720 USA; 2 College of Tropical Agriculture and Human Resources, Komohana Research and Extension Center, University of Hawai’i, Hilo, HI 96720 USA; 3 USDA-ARS, Tropical Pest Genetics and Molecular Biology Research Unit, Daniel K Inouye US Pacific Basin Agriculture Research Center, Hilo, HI 96720 USA

**Keywords:** Microbiome, *Bactrocera dorsalis*, *Klebsiella*, *Providencia*, Gut, Ovipositor, 16S rRNA

## Abstract

Bacterial symbionts are crucial to the biology of *Bactrocera dorsalis*. With larval diet (fruit host) being a key factor that determines microbiome composition and with *B. dorsalis* using more than 400 fruits as hosts, it is unclear if certain bacterial symbionts are preserved and are passed on to *B. dorsalis* progenies despite changes in larval diet. Here, we conducted a fly rearing experiment to characterize diet-induced changes in the microbiome of female *B. dorsalis*. In order to explicitly investigate the impacts of larval diet on the microbiome, including potential stable bacterial constituents of *B. dorsalis*, we performed 16S rRNA sequencing on the gut tissues of teneral female flies reared from four different host fruits (guava, mango, papaya, and rose apple) infested using a single cohort of wild *B. dorsalis* that emerged from tropical almond (mother flies). Although *B. dorsalis*-associated microbiota were predominantly shaped by the larval diet, some major bacterial species from the mother flies were retained in progenies raised on different larval diets. With some variation, *Klebsiella* (ASV 1 and 2), *Morganella* (ASV 3), and *Providencia* (ASV 6) were the major bacterial symbionts that were stable and made up 0.1–80% of the gut and ovipositor microbiome of female teneral flies reared on different host fruits. Our results suggest that certain groups of bacteria are stably associated with female *B. dorsalis* across larval diets. These findings provide a basis for unexplored research on symbiotic bacterial function in *B. dorsalis* and may aid in the development of novel management techniques against this devastating pest of horticultural importance.

**Supplementary Information:**

The online version contains supplementary material available at 10.1007/s00248-024-02352-9.

## Introduction

Bacterial symbionts can have crucial roles in the success of insects in their environment. They share common niches with insects and often engage in intricate ecological interactions that influence an insect’s overall fitness [1] by affecting their growth, fecundity, lifespan, and mate selection [2–4]. Additionally, bacteria may also mediate interactions with infectious disease and natural enemies [5, 6], impart temperature stress tolerance [7, 8], aid in the metabolism of xenobiotics [9, 10], and provide nutrients to their host insects [11, 12].

Bacterial symbionts can generally exist as obligate and facultative symbionts [13]. Obligate symbionts are essential for the host insect’s survival [14] and enable insects to survive adverse conditions [15], and in return, the host insect provides shelter and supplies nutrients to its bacterial partner [16]. Such symbiosis are cases of obligate symbiosis, as bot, the insect and its symbiotic bacteria suffer and even perish in the absence of each other [17]. Obligate symbionts are most often transmitted vertically from mother to offspring [18]. In contrast, facultative symbionts are not essential for the survival of the host insect but aid their hosts under certain conditions and are transmitted vertically or horizontally to the host’s offspring [13, 19]. An example of facultative symbionts is commensal bacteria, which can also endure in various niches in the absence of the host insect [20]. Previous studies have provided convincing evidence that commensal bacteria, despite being non-essential for the survival of host insect, contribute to important aspects of their host insect’s biology [3, 4, 7]. Thus, a thorough understanding of host-microbiome interactions also requires comprehensive knowledge of acquisition, transmission, host-associated bacterial communities, and the factors shaping them.

The acquisition and transmission of bacterial symbionts and pathogens in *B. dorsalis* often occur through contact with substrates with bacterial presence, such as fruit/leaf surfaces. However, the majority of bacterial symbionts are acquired within the larval feeding environment (host fruit or diet) that contains microbes deposited by the mother fly during oviposition. Although some studies have explored the dynamics of microbial communities in *B. dorsalis* [21], studies on bacteria that get retained by *B. dorsalis* even with the change of larval diet are limited.

Microbial communities in insects can be influenced by many factors with “larval diet” being a major contributor [21]. To explore factors shaping insect microbial communities, researchers often studied the association between host genetic divergence and diet. For example, the genetic correlation between host taxa and variation in the microbial community may suggest the role of genetic effects in shaping insect microbial communities, while the correlation between diet and microbial community composition may point to environmental effects. Although this approach has been applied in vertebrate systems [22–24], the factors that determine the microbiome composition in higher animals are not yet clear. This is partly due to the difficulty of controlling diet and other environmental factors and the complexity of microbial communities associated with vertebrates [25–27], which consist of thousands of taxa. In contrast, the gut microbiome of invertebrates, especially insects, is comprised of substantially fewer bacterial taxa [28, 29]. This makes insects as attractive models for disentangling complex host-microbial interactions [29, 30]. Furthermore, insects can serve as axenic and gnotobiotic models, and such experiments can widen our knowledge on insect-microbe interactions [31, 32].

The oriental fruit fly, *Bactrocera dorsalis* (Diptera: Tephritidae) is an important insect pest of horticultural crops around the globe [33]. This insect causes significant economic losses from fruit damage and export limitation due to quarantine issues [34–36]. Additionally, its utilization of a broad host range, temperature tolerance, and high mating success makes it a serious pest with extreme invasive potential [37–39], and these abilities may in part be attributed to their microbiome [4, 7]. Recently, there is growing evidence that there are key metabolic roles associated with bacterial symbionts found in a tephritid fly’s microbiome, and specific bacterial taxa may gain an advantage over others in different host fruits [21, 40, 41]. However, there is a possibility that more than one kind of bacterial taxa can fulfill a particular metabolic role (phenol degradation, cellulose degradation, etc.), thus prompting some plasticity in the microbiome composition of flies that emerged from different host fruits [21].

To understand the diversity and potentially stable constituents of bacterial communities associated with *B. dorsalis*, it is imperative to investigate teneral flies from different host fruits (larval diet) that have a known common wild fly mother. Therefore, we investigated the microbiome of teneral female flies from four different host fruits that were infested by a single cohort of wild flies that emerged from tropical almond (mother flies).

## Materials and Methods

### Collection and Rearing of Insects

**Fig. 1:**
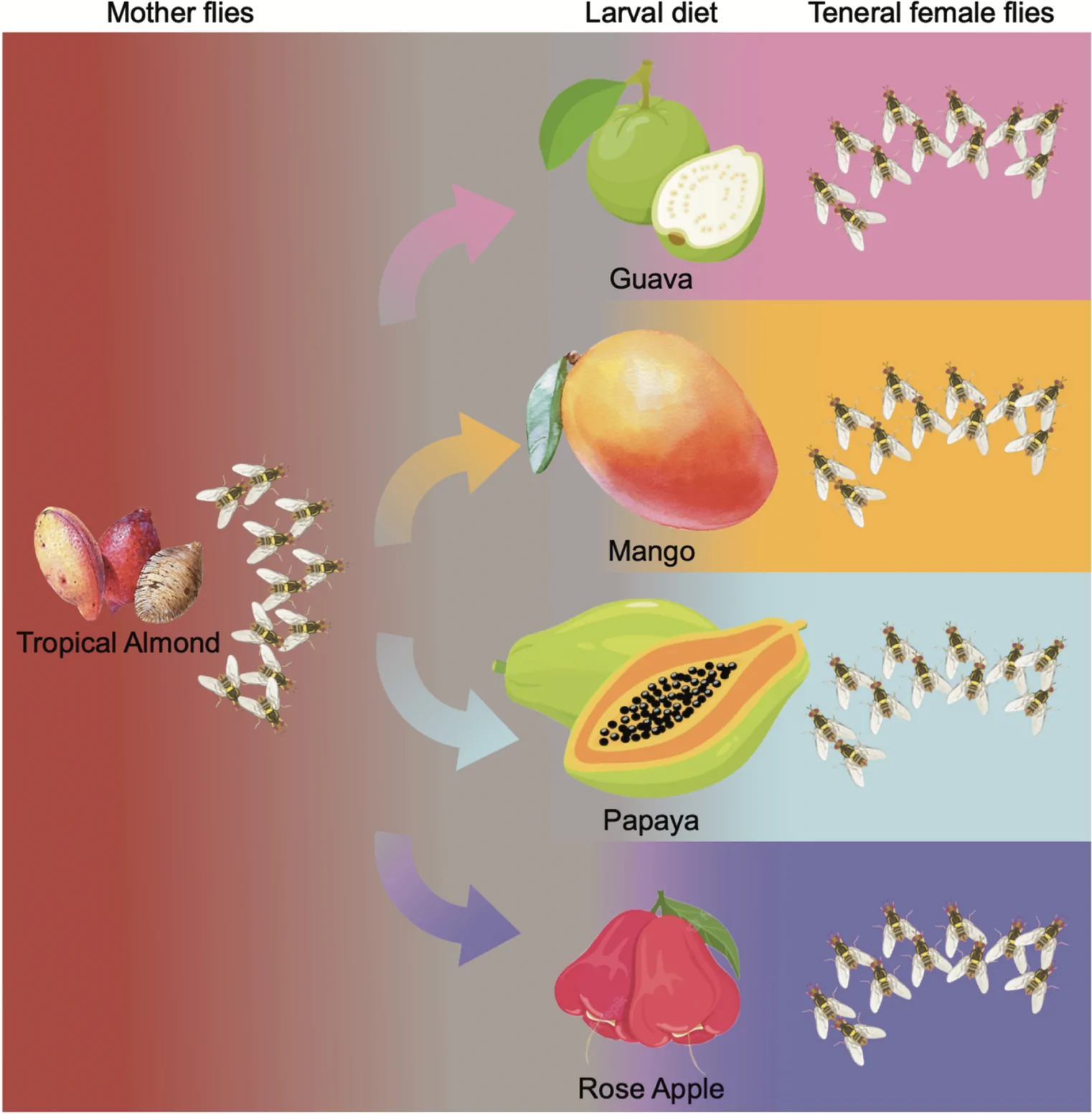
Graphical representation of the experimental design. Mother flies were sourced from infested tropical almonds. A single cohort of mother flies from tropical almonds was allowed to oviposit into four different fruit hosts (larval diet; guava, mango, papaya, and rose apple). The teneral female flies that emerged from each fruit host were further used to study their gut and ovipositor microbiome

The mother culture of *B. dorsalis* was obtained from tropical almond fruits collected from Onekahakaha Beach Park, Hilo, HI, USA, in June–July 2022. Infested tropical almond fruits were transferred to the laboratory (25 ± 1 °C, 65% RH, and 16:8 h light and dark photoperiod) and placed on a 2-cm thick layer of sterile vermiculite (Vigoro, USA) to aid pupation. Pupae were sieved from the sterile vermiculite into a sterile Petri plate (90 mm dia.) and placed in rearing cages (30 × 30 × 30 cm, BugDorm.com) for adults to emerge. Emerged adult flies were provided with a mixture of honey and yeast hydrolysate (1:1 w/w) and water ad libitum for maturation and egg development. Mature gravid female flies (30 days old) from tropical almond fruits were provided with either guava, mango, papaya, or rose apple separately as oviposition substrates (Fig. 1). Organic fruits from local grocery stores were washed with a diluted soap solution, followed by 5% sodium hypochlorite solution for 1 min and rinsed in distilled water three times, and wiped dry using sterile tissues before providing them to wild *B. dorsalis* gravid female flies that emerged from tropical almond fruits (30 days old; 50 flies). After oviposition occurred (48 h), each fruit was placed into individual containers holding sterile vermiculite (2-cm thick layer) to aid pupation. The pupae that developed from the individual fruits were placed separately in rearing cages, and the newly emerged teneral female flies were used for further experiments.

### Dissection of Gut Tissues of Teneral Flies

Tissues of flies from tropical almond (mother flies; 30 days old), guava, mango, papaya, and rose apple (teneral female flies;1 day old; not fed with diet) were dissected using general aseptic dissection procedures from gut and ovipositor. Prior to dissection, female flies from each fruit were kept at 5 °C for 15 mins to slow down the movement and were individually surface sterilized with 5% sodium hypochlorite solution for 30 s followed by a wash with 70% ethanol for 1–2 min and then rinsed with sterilized distilled water for 3 min. Female flies (*n* = 10 per host fruit species) were dissected individually on an aseptic plate containing 2 ml sterilized phosphate-buffered saline (PBS, 137 mM NaCl, 2.7 mM KCl, 10 mM Na_2_HPO_4_, 2 mM KH_2_PO_4_, pH 7.4) using sterilized forceps under a stereomicroscope. The forceps were sterilized intermediately during the dissection procedure by dipping in ethanol (70%) and flaming the tips using a spirit lamp. The whole gut tissue including the ovipositor was dissected, and later, the ovipositor along with the oviduct was separated from the whole gut tissue. Gut and ovipositor tissues from each fly were transferred separately into individual aseptic centrifuge tube containing 1 ml of DNA/RNA Shield (Zymo Research), thus making 10 replicates of tissues from flies that emerged from each selected fruit. The tubes containing sample tissues were stored at −80 °C until further use.

### DNA Extraction and Microbiome Analysis

DNA was extracted using a ZymoBIOMICS DNA Extraction kit on a KingFisher Flex Magnetic Particle Processor (Thermo Scientific). Mock communities (Zymo) and empty wells were included in the extraction plates as positive and negative controls, respectively. 16S-rRNA metabarcoding was conducted as described previously [42]. The V4 sub-region of the 16S SSU rRNA was amplified using 515F (GTGCCAGCMGCCGCGGTAA) and 806R (GGACTACHVGGGTWTCTAAT). Reactions were performed in 25 µl volumes using Q5 Hot Start High-Fidelity Polymerase (New England Biolabs) with 0.2 µM of each primer. Reaction conditions were 98 ℃ for 30 s, 30 cycles of 98 ℃ for 30 s, 50 ℃ for 30 s, and 72 ℃ for 2 min, and a final extension at 72 ℃ for 10 min. PCR products were normalized using a Just-A-Plate normalization kit (Charm Biotech). Amplicon pools were sequenced at the ASGPB Genomics Core at the University of Hawai’i at Manoa using Illumina MiSeq V3 600 chemistry (Illumina, San Diego, CA, USA).

### Processing of Sequence Data

Illumina 16S reads were processed with the “DADA2” (v. 1.24) pipeline to obtain amplicon sequence variants (ASVs) [43] implemented in R. Steps included filtering, dereplication, inference of sequence variants, mergers of paired-end reads, and chimera detection and removal. Taxonomy assignments were performed using “DECIPHER” (v. 2.24.0) [44] with a trained version of the Ribosomal Database Project (RDP) reference database (v 18) and ASVs assigned to chloroplast and mitochondria, or those which were unclassified at the domain level were removed from the dataset prior to statistical analyses. After initial processing and taxonomic assessment, we performed classifications of ASVs with the RDP naïve Bayesian classifier with the v18 database [45].

### Statistical Analyses

Statistical analyses were performed using R 4.2.1 in RStudio [46, 47]. Microbiome data were analyzed using “vegan” [48] after processing with DADA2 [43]. Samples were rarefied to 8000 sequences for downstream analyses. ASV composition and membership were analyzed using Bray-Curtis and Jaccard distances, respectively. Distances were visualized and analyzed using non-metric multidimensional scaling (NMDS) and PERMANOVA implemented with adonis2. Since gut and ovipositor samples were from paired individuals, we used individuals as strata in the model. NMDS plots were made for the individual tissues (gut and ovipositor) to display differences in the initial fruit source. Pairwise multivariate analysis of Bray-Curtis and Jaccard distances was performed using the package “pairwiseAdonis” using a false discovery rate (FDR) correction [49]. Diversity metrics such as ASV richness, Shannon, and 1/Simpson metrics were computed in vegan and analyzed using a Kruskal-Wallis test with pairwise comparisons being performed with Dunn tests [50]. In order to identify potential core components of the oriental fruit fly microbiomes in our study, we evaluated occupancy-abundance distributions of the ASVs associated with the flies. ASVs were grouped by the individual fly specimen (gut and ovipositor averaged). When applying a stringent occupancy = 1, we observed no conserved ASVs across all individual samples. Therefore, we conducted occupancy-abundance analysis using a threshold based on contribution to Bray-Curtis similarity [51]. The 500 most prevalent ASVs were ranked according to their contribution to Bray-Curtis beta-diversity, with a cutoff of core ASVs by the last 2% increase in explanatory value by Bray-Curtis similarity [51]. Both core and ASVs were plotted against a Sloan neutral model constructed using the R package “tyRa” [51–53]. Heatmap of core ASVs was generated using “pheatmap” [54], with Bray-Curtis distances being used to cluster samples and visualization of ASV relative abundances with a Z-transformation of ASV relative abundances.

## Results

### Sequencing of Controls

We included a negative kit control and positive mock communities through our extraction procedure and performed PCR alongside our experimental samples. The negative control yielded no sequences at the end of the pipeline. Sequencing of positive controls returned ASV numbers and taxonomical classifications to what was expected from the mock community (Fig. S1). Eight ASVs comprised >99% of the sequences in the mock community controls. Relative abundances of controls in the two extraction plates did not exhibit any marked differences from each other. There was some variation in the processed controls from the theoretic makeup, but that is unexpected given the biases inherent in PCR-based approaches.

### Host Fruit Influences Microbial Community Composition

**Fig. 2:**
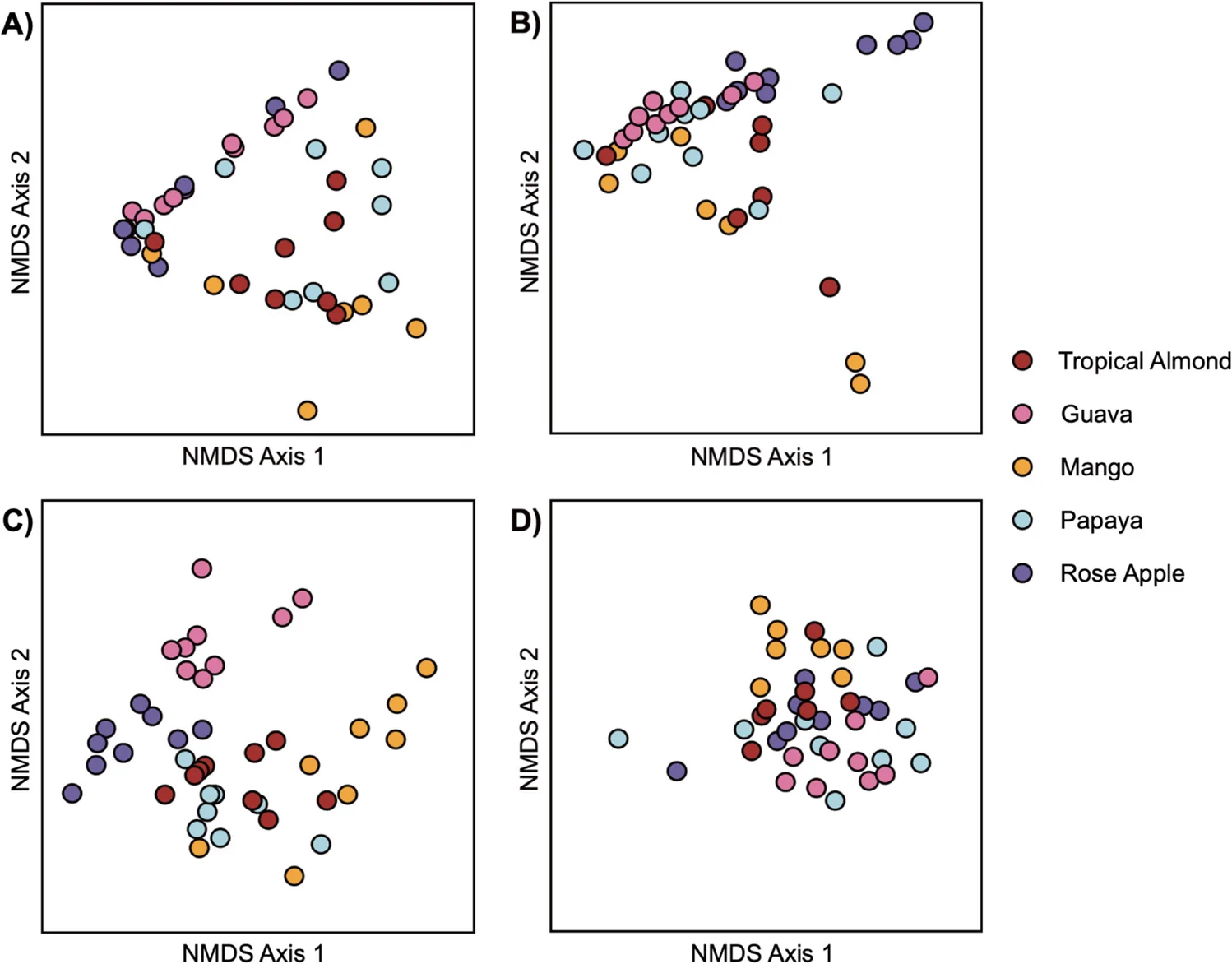
NMDS plots showing the impact of fruit source on female *B. dorsalis* 16S ASVs. Gut samples (**A**, **C**) and ovipositor samples (**B**, **D**) were analyzed separately for these analyses. NMDS plots were constructed with Bray-Curtis (top) and Jaccard (bottom) dissimilarities. Plots indicate some clustering of samples from different fruit sources, especially with gut tissues

Larval diet had effects on bacterial ASV composition associated with *B. dorsalis* guts and ovipositors. Using Bray-Curtis distances observed, there were effects of larval diet (fruits) (PERMANOVA-*F*_4,82_ = 4.95, *p* < 0.001), but not the host tissue (*F*_1,82_ = 1.75, *p* = 0.122). We did observe interactive effects between host fruit and tissue source (*F*_4,82_ = 2.43, *p* = 0.002). Evaluating pairwise comparisons, most of the paired gut and ovipositor samples have similar responses with a couple of exceptions (Table S1). In all but one case (rose apple), there were no differences in gut and ovipositor memberships (*p* > 0.05). NMDS plots of separated gut and ovipositor samples demonstrated a triangular shape in the ordination (Fig. 2A, B) as well as some separation of samples originating from different host fruits, suggesting that the hosts yielded three different dominant ASV memberships. There were overlapping individuals from different host plants in both cases, indicating that, while the larval diet has an impact on the ASV composition, there is variation among the treatments. Jaccard distances using presence/absence data indicated additional differences in ASV memberships (Fig. 2C, D). PERMANOVA of Jaccard distances indicated that there was a significant impact of larval diet (*F*_4,82_ = 3.38, *p* < 0.001), adult tissue type (*F*_1,82_ = 5.91, *p* < 0.001), and their interaction (*F*_4,82_ = 1.84, *p* < 0.001) on ASV membership among samples. Compared to Bray-Curtis dissimilarities, pairwise Jaccard distances yielded far more differences between individuals, suggesting that sparsely populated aspects of the microbiome diverged between the female tissues. Additionally, Jaccard plots demonstrated a higher degree of structuring by host plant in both gut (Fig. 2C) and ovipositor (Fig. 2D) samples, indicating greater contribution of host plants to less-dominant aspects of the *B. dorsalis* adult female microbiome.

**Fig. 3:**
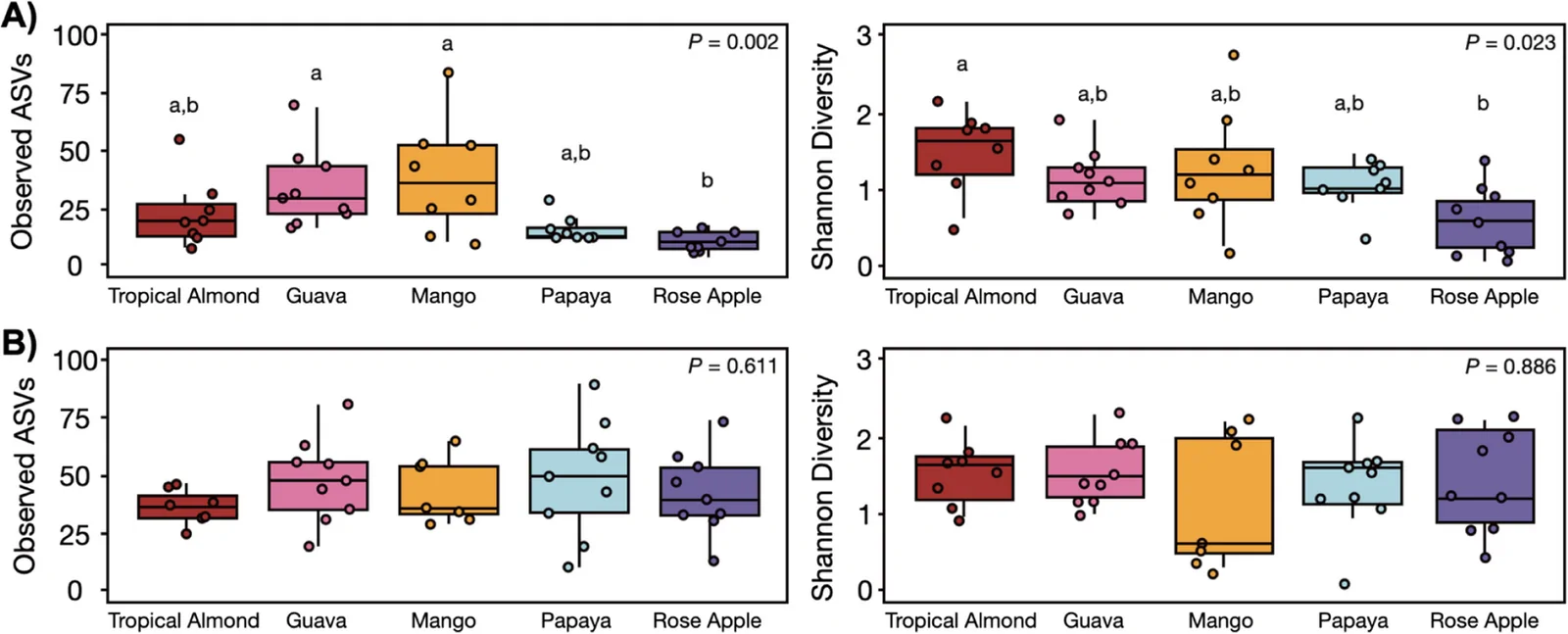
ASV richness and Shannon diversity of *B. dorsalis* gut (**A**) and ovipositor (**B**). Boxplots reflect the median and interquartile range, with individual points representing unique samples

Influence of larval diet on *B. dorsalis* ASV richness and diversity estimates analysis varied between tissue types (Fig. 3). For gut tissues, there was a significant effect of host plant on ASV richness (Fig. 2A; χ^2^ = 16.7, *p* = 0.002) and Shannon diversity (χ^2^ = 11.3, *p* = 0.023). Gut samples (Fig. 3A) collected from flies originating from rose apple reduced ASV richness than those from guava and mango and had less diversity than specimens collected from tropical almond. *Bactrocera dorsalis* ovipositor samples (Fig. 3B) did not differ in either richness (χ^2^ = 2.6, *p* = 0.627) or diversity (χ^2^ = 1.2, *p* = 0.886).

### Evaluating Incidence of ASVs Across Samples

**Fig. 4:**
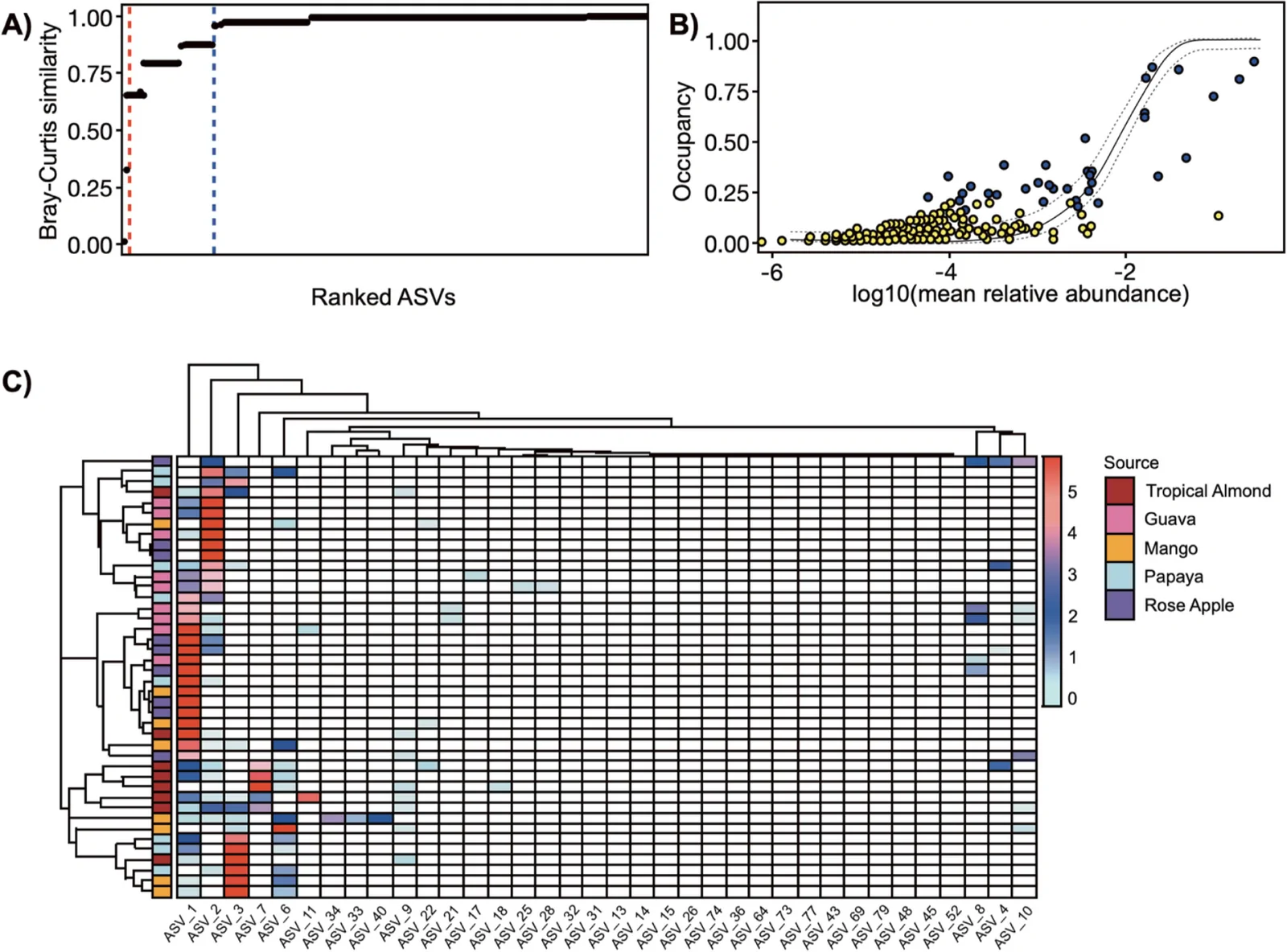
Determination and distribution of *B. dorsalis* ASVs as determined by occupancy-abundance analysis across all individual samples. Three core ASVs were determined using first-order differences (red line), and 36 were by contributions to Bray-Curtis distances (blue line) (**A**). Occupancy-abundance plot (**B**) displaying the 200 most prevalent ASVs across all samples, where an occupancy of 1 indicates presence across all sample types. ASVs that are shaded blue are those determined to be core components according to the Bray-Curtis cutoff method, with ASVs mapped to the Sloan neutral model. Heatmap (**C**) was constructed using only gut microbiome samples, with sample clustering being performed using Bray-Curtis distances between sample types and ASVs clustered using Euclidean distances. ASV relative abundances were z-score transformed before plotting

We used occupancy-abundance relationships using a Bray-Curtis similarity cutoff to evaluate potentially core ASVs across sample types (Fig. 4). A first-order cutoff (Fig. 4A; red line) yielded three potentially core ASVs, while 31 ASVs were determined to contribute to Bray-Curtis dissimilarity above a 2% cutoff (Fig. 4A; blue line). None of these core ASVs were present across all individuals sampled (Fig. 4B), with the highest incidence being ~85%. However, despite these ASVs not having universal occupancy, they were present in a specimen from each of the larval host fruits (Fig. 4C). Heat map clustering of the dominant ASVs in gut samples also demonstrated the sparseness of the *B. dorsalis* adult fly microbiome, with ~10 ASVs being the most dominant. As suggested by the ordination analysis, the gut samples were clustered into three main groups (left axis), generally following patterns indicated by host fruits. Samples were generally dominated by two to five ASVs, with the remaining community having relatively low abundances.

### Taxonomic Consistency Across the Samples

**Fig. 5:**
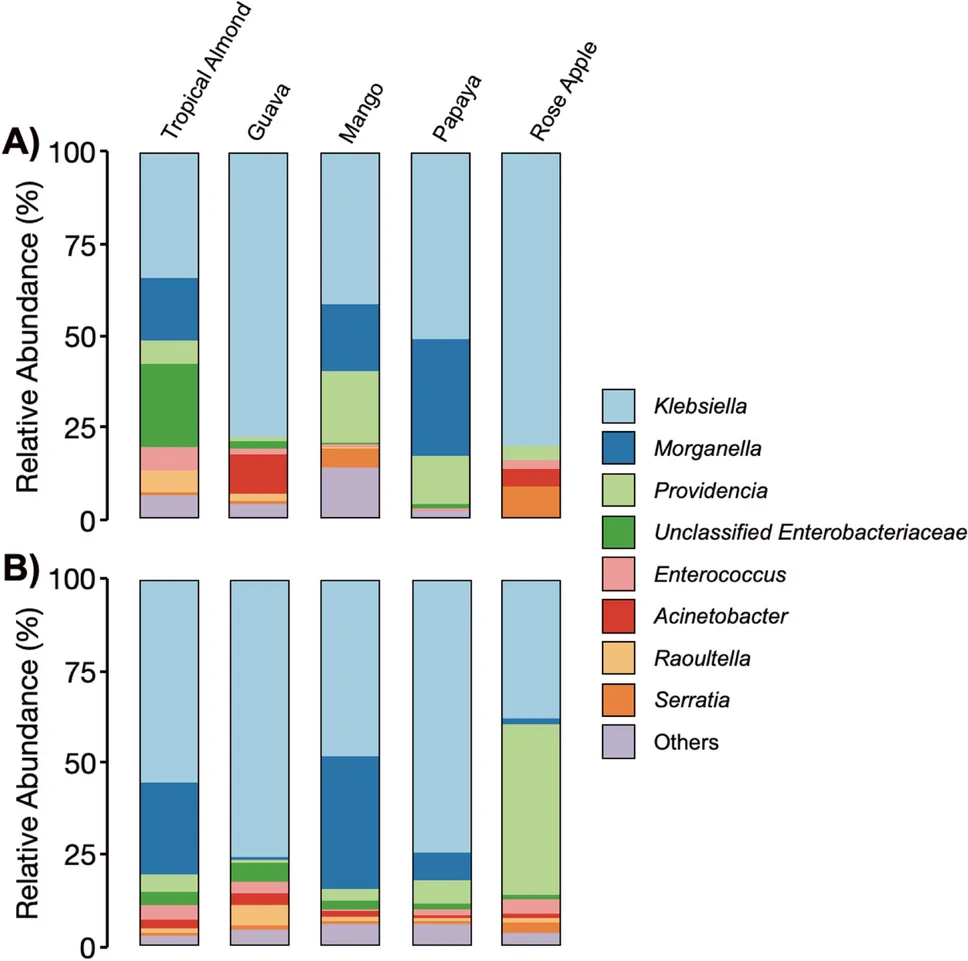
Relative abundance (%) of ASVs associated with *B. dorsalis* gut (**A**) and ovipositor (**B**). Classifications were conducted using the RDP classifier (v 2.13) using the v.18 training set. Taxonomy was determined at a 60% bootstrap threshold. Taxa with relative abundances < 2% across all specimens were grouped into the “Other” category

The three most prevalent ASVs in gut samples were classified as *Klebsiella* (ASV1 and 2), *Morganella* (ASV3), and *Providencia* (ASV6) (Fig. 4C). Because the ASVs classified as *Klebsiella* exhibited unique colonization patterns, it suggests that these are likely originating from separate strains that differentially associated with the samples. Evaluating the relative abundances of ASV taxa, we saw similar patterns. Overall, our results show that, on the genus level (Fig. 5A, 5B), the microbiome of flies from all fruits and tissue was dominated by *Klebsiella* (34–80%). Similarly, *Providencia* (0.15–48%) was also present in the gut and the ovipositor of flies from all selected fruits. In contrast, *Morganella* was present in the gut of flies from tropical almond (25%), mango (36%), and papaya (8%) but was virtually absent in guava and rose apple. However, they were present in the ovipositor of flies from all the fruits. The remaining genera, namely, unclassified *Enterobacteria* (0.6–23%), *Enterococcus* (0.8–6%), *Acinetobacter* (0–11%), *Raoultella* (0–6%), and *Serratia* (0–8%) were present in low abundance. It should be noted that although the major bacterial constituents in flies were similar, the overall abundance of other bacterial genera, in both gut and ovipositor of flies from guava, mango, papaya, and rose apple, differed from the microbiome of the mother culture (flies from tropical almond). It is also interesting to note that the three major taxa, *Klebsiella*, *Providencia*, and *Morganella*, dominated the microbiome and likely influenced other minor bacterial taxa. We could clearly see that the major bacterial taxa (*Klebsiella*, *Providencia*, and *Morganella*) replaced each other but did not allow other bacterial taxa to replace them although the flies emerged from different fruits (larval diet). This evidently proves that these three major taxa are crucial and may have an influence on the microbiome structure to some extent, other than the diet. However, further experiments are needed to confirm this aspect.

## Discussion

Insect guts and ovipositors are complex environments where host and environmental factors can collectively influence microbial composition and function. *Bactrocera dorsalis* harbors a complex microbiome that affects its behavior and nutritional status [55–57]. In our study, we found high variability in the microbiome composition between individuals, but we found some bacterial ASVs and corresponding genera present in high frequency in all the individual flies tested. Similarly, in *Drosophila*, the microbiome composition can be highly variable between individuals [58], though some key bacterial species are typically present in higher frequency [29]. The goal of our study was to identify potential stable constituents of the *B. dorsalis* female fly microbiome.

We determined that the microbiome structure varied between individual teneral flies raised on different host fruits and between individual flies raised on the same fruit suggesting that the forces influencing the microbiome structure extend beyond diet. However, we found ASVs taxonomically classified as *Klebsiella* present in all teneral female flies irrespective of their origin, though at different relative abundances (Fig. 4). This supports other studies from all around the globe that have indicated *Klebsiella* to be an important aspect of tephritid fruit flies’ microbiome [[59] and references within]. ASVs/OTUs of Enterobacteriaceae [60–63] and particularly *Klebsiella* are highly dominant taxa [64–66] in most of the tephritid flies. *Klebsiella* spp. have been shown to improve larval development and can affect adult performance and behavior, aiding courtship and reproduction in tephritid flies [67–70]. They may also contribute to the fly fitness by increasing nitrogen availability through nitrogen fixation, an common trait of *Klebsiella* [71, 72]. *Klebsiella* is also known to provide carbon to the flies by pectinolysis, a trait of *K. oxytoca* [73], a dominant cultured bacterial species in tephritid fruit flies including *B. dorsalis* [3, 7, 74, 75]. This suggests that *Klebsiella* may have some integral functions in the life history of fruit flies and warrants further exploration.

The microbiome of the gut and ovipositor of flies raised on selected fruits was generally consistent except for the microbiome of flies raised on rose apple that was dominated by *Providencia*. However, *Providencia* was abundant in the gut (10–40% of relative abundance) in all teneral flies. *Providencia* is a gram-negative, non-spore forming bacteria that is an opportunistic pathogen to some insects [75] and has also been isolated from many fruit fly species including *A. ludens* [76, 77], *A. obliqua*, *A. serpentina*, *A. striata* [77], *B. dorsalis* [3], *B. oleae* [31], *B. tryoni* [78], *Ceratitis capitata* [79], *Zeugodacus tau* [80], *Z. cucurbitae* [64], and *Drosophila melanogaster* [81]. To date, there is no evidence of these bacteria causing infections in *B. dorsalis* including our unpublished data from colonies maintained by the USDA ARS (Mason unpublished) and feeding experiments (Kempraj and Cha unpublished). On the contrary, some studies have demonstrated positive effects of *Providencia* on the development of fruit flies and improving fly resistance to fungal infection [31]. The other bacterial taxa that were abundant in the flies’ gut and ovipositor tissues were *Morganella*. Salas et al. [82] observed *M. morganii* to cause infection in reared *A. ludens* larvae and was also detected in wild *B. dorsalis* [3], *B. tryoni* [78], and *Z. tau* [80]. Although potentially pathogenic, the reason for the abundance of these bacteria in the gut and ovipositor tissues in teneral flies is to be uncovered.

In our analysis, we observed some differences in how host fruit altered the ovipositor microbiome compared to that of the gut. Currently, we can only speculate what may drive these differences. Considering that the samples were surface sterilized, we do not think that external colonizers were the drivers of these differences. We suspect that differences in tissue structure may be the root cause. The ovipositor and junction between the gut and ovipositor are comprised of a cuticle, while the epithelial cells are lined with a mucosal-like peritrophic matrix. These substrates can favor different colonization frequencies and result in relatively distinct microbial communities for insects [83, 84]. Additionally, the gut is active and dynamic in regulating microbial populations where the ovipositor structures may not be. Further manipulative experiments are needed to understand these differences for *B. dorsalis* and other fruit flies.

The present study explored differences in the gut and ovipositor microbiome of teneral *B. dorsalis* females reared from wild origins on four different host fruits. Overall, we found that the larval diet does have an effect on the gut microbial composition of newly emerged flies. However, we also found that some bacterial genera, such as *Klebsiella*, *Providencia*, *and Morganella*, that were abundant in the wild mother fly were also abundant and preserved in the flies that were reared on different host fruits. This result is similar to other studies on humans and *Drosophila*, where different individuals could preserve and maintain specific microbes even after extensive dietary changes [85, 86]. Taken together, our results show that although there is a possibility of vertical transfer of specific bacterial constituents from the mother to progeny, larval diet (host fruit) had a profound impact on the microbiome structure overall [21]. It would be safe to consider that the genera *Klebsiella*, *Providencia*, and *Morganella* are the major bacterial constituents that are extremely stable in flies from all the selected fruits, but there are potentially different strains that could dominate the gut microbiome. This raises questions about how similar bacterial strains may compete and dominate in the guts of insects. Further exploration with other sequencing techniques alongside manipulative experiments is needed to determine the ecological drivers and ultimate effects. Overall, the knowledge provided here can aid us to understand *B. dorsalis*-microbiome interaction in an ecological context and shows that for stable interactions, the fly may have to conserve a few specific bacteria to possibly increase fitness. Moreover, these results develop the use of *B. dorsalis* as a model to study microbiota proliferation and colonization. Upon understanding the role of these specific bacteria in the life history of *B. dorsalis*, the knowledge may aid us to develop novel management strategies to control this devastating horticultural pest.

## Supplementary Information

Below is the link to the electronic supplementary material.

Supplementary file1 (PDF 201 KB)

## Data Availability

Data and R code has been deposited in public databases. Raw sequence reads are available at NCBI SRA (PRJNA1061202). Processed ASV sequences, tables, taxonomy, and associated R code have been deposited at USDA ARS Ag Data Commons  (10.15482/USDA.ADC/24969123)
