# Supplementary material for: Impact of Larval Food Source on the Stability of the Bactrocera dorsalis Microbiome
Source: Microb Ecol. 2024 Feb 26;87:46. doi: 10.1007/s00248-024-02352-9 (PMC10896919; doi:10.1007/s00248-024-02352-9)
Supplement: Supplementary file 1 [file 248_2024_2352_MOESM1_ESM.pdf]

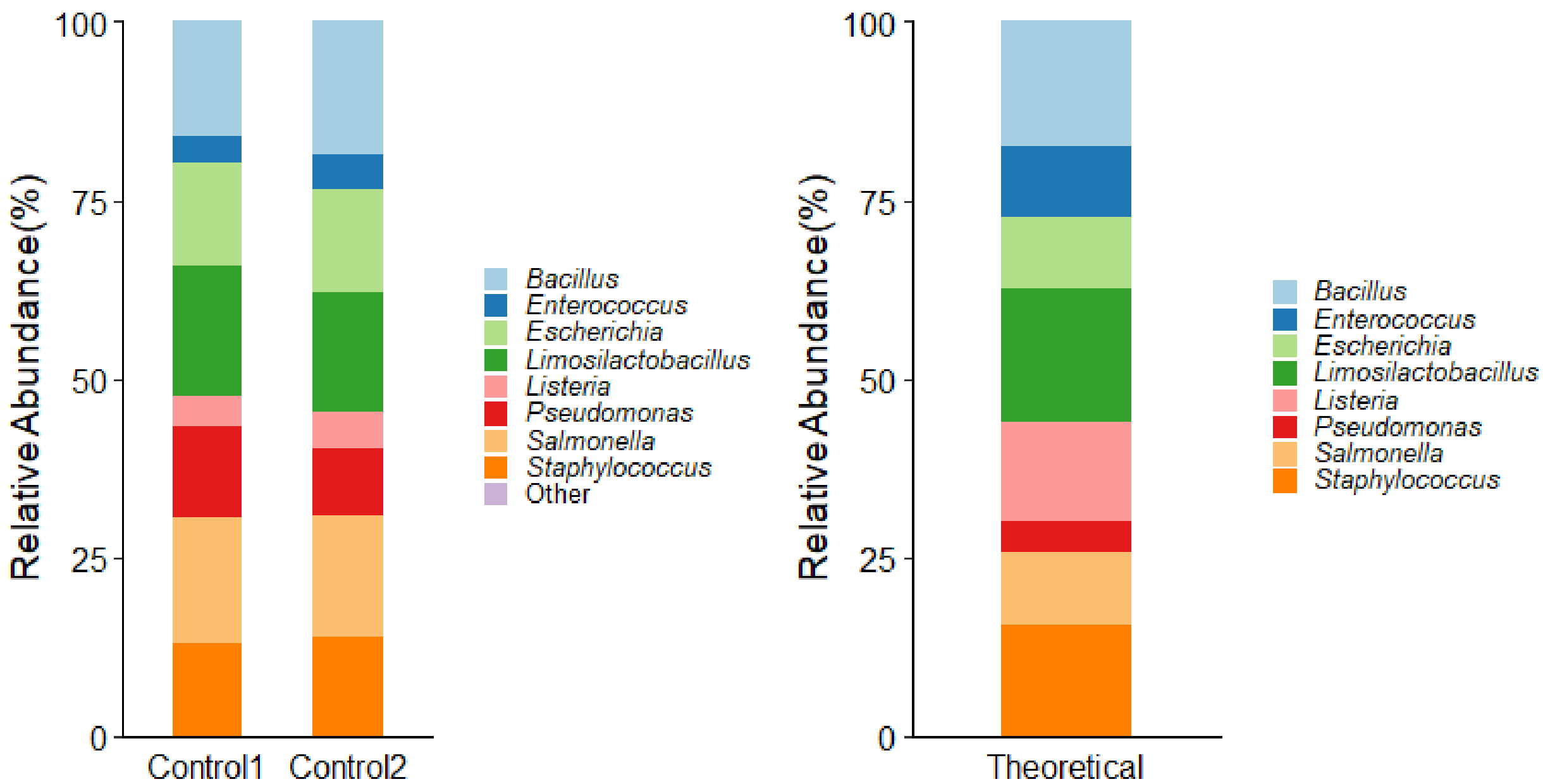


Supplementary Figure 1: Performance of positive microbial extraction control compared to theoretical makeup. 8 ASVs comprised > 99% of the sequences.

Supplementary Table 1: Pairwise PERMANOVA comparisons comparing tissue types across different fruits.

A. Bray-Curtis Comparisons:

| | Guava Ovipositor | Mango Gut | Mango Ovipositor | Papaya Gut | Papaya Ovipositor | Rose Apple Gut | Rose Apple Ovipositor | Tropical Almond Gut | Tropical Almond Ovipositor |
|---|---|---|---|---|---|---|---|---|---|
| Guava Gut | 0.339 | **0.008** | **0.009** | **0.021** | 0.193 | 0.334 | **0.009** | **0.008** | 0.068 |
| Guava Ovipositor | | **0.011** | **0.008** | **0.019** | 0.193 | 0.193 | **0.008** | **0.014** | 0.068 |
| Mango Gut | | | 0.275 | 0.232 | **0.050** | **0.035** | **0.008** | **0.050** | 0.099 |
| Mango Ovipositor | | | | 0.193 | **0.027** | **0.035** | **0.008** | 0.078 | 0.256 |
| Papaya Gut | | | | | 0.152 | **0.049** | **0.014** | **0.041** | 0.367 |
| Papaya Ovipositor | | | | | | 0.193 | **0.009** | **0.011** | 0.225 |
| Rose Apple Gut | | | | | | | **0.014** | **0.014** | 0.080 |
| Rose Apple Ovipositor | | | | | | | | **0.009** | **0.020** |
| Tropical Almond Gut | | | | | | | | | 0.193 |

B. Jaccard Comparisons

| | Guava Ovipositor | Mango Gut | Mango Ovipositor | Papaya Gut | Papaya Ovipositor | Rose Apple Gut | Rose Apple Ovipositor | Tropical Almond Gut | Tropical Almond Ovipositor |
|---|---|---|---|---|---|---|---|---|---|
| Guava Gut | **0.002** | **0.001** | **0.001** | **0.001** | **0.001** | **0.001** | **0.001** | **0.001** | **0.001** |
| Guava Ovipositor | | **0.001** | **0.001** | **0.001** | **0.012** | **0.001** | **0.001** | **0.001** | **0.001** |
| Mango Gut | | | **0.001** | **0.001** | **0.001** | **0.001** | **0.001** | **0.001** | **0.001** |
| Mango Ovipositor | | | | **0.001** | **0.001** | **0.002** | **0.001** | **0.001** | **0.001** |
| Papaya Gut | | | | | **0.001** | **0.001** | **0.001** | **0.001** | **0.001** |
| Papaya Ovipositor | | | | | | **0.001** | **0.05** | **0.001** | **0.02** |
| Rose Apple Gut | | | | | | | **0.001** | **0.001** | **0.001** |
| Rose Apple Ovipositor | | | | | | | | **0.001** | 0.307 |
| Tropical Almond Gut | | | | | | | | | **0.013** |

Supplementary Table 2: Wilcoxon rank sum tests comparing ASVs of different host tissues across different fruits. Table shows statistically significant ASVs. Numbers show relative abundance (%).

| | ASV | P-Value | Gut Rel Abund | Ovipositor Rel Abund | Taxon |
|---|---|---|---|---|---|
| Guava | ASV107 | 0.029 | 0.001 | 0.017 | Lactococcus |
| | ASV11 | 0.012 | 1.732 | 2.299 | Raoultella |
| | ASV117 | 0.029 | 0.000 | 0.016 | Burkholderia |
| | ASV15 | 0.006 | 0.066 | 0.130 | Staphylococcus |
| | ASV29 | 0.024 | 0.208 | 0.033 | Klebsiella |
| | ASV3 | 0.001 | 12.420 | 12.107 | Morganella |
| | ASV31 | 0.002 | 0.094 | 0.805 | Klebsiella |
| | ASV45 | 0.030 | 0.021 | 0.071 | Dermacoccus |
| | ASV6 | 0.009 | 6.190 | 3.537 | Providencia |
| | ASV7 | 0.010 | 4.264 | 1.222 | Unclassified Enterobacteriaceae |
| Mango | ASV10 | 0.015 | 2.889 | 1.188 | Serratia |
| | ASV101 | 0.017 | 0.000 | 0.020 | Pseudomonas |
| | ASV127 | 0.002 | 0.000 | 0.025 | Pseudoxanthomonas |
| | ASV150 | 0.006 | 0.000 | 0.020 | Frateuria |
| | ASV165 | 0.047 | 0.000 | 0.018 | Pseudomonas |
| | ASV166 | 0.017 | 0.000 | 0.013 | Novosphingobium |
| | ASV181 | 0.016 | 0.006 | 0.000 | Unclassified Saccharibacteria_genera_incertae_sedis |
| | ASV24 | 0.038 | 0.838 | 0.000 | Flavobacterium |
| | ASV32 | 0.007 | 0.223 | 0.073 | Fructobacillus |
| | ASV43 | 0.026 | 0.007 | 0.169 | Pantoea |
| | ASV49 | 0.016 | 0.127 | 0.002 | Chryseobacterium |
| | ASV53 | 0.038 | 0.089 | 0.000 | Variovorax |
| | ASV62 | 0.038 | 0.062 | 0.000 | Caulobacter |
| | ASV8 | 0.002 | 3.051 | 0.943 | Acinetobacter |
| | ASV85 | 0.038 | 0.022 | 0.000 | Flavitalea |
| | ASV87 | 0.038 | 0.024 | 0.000 | Rhodopseudomonas |
| Papaya | ASV10 | 0.004 | 2.889 | 1.188 | Serratia |
| | ASV100 | 0.038 | 0.000 | 0.016 | Enhydrobacter |
| | ASV101 | 0.017 | 0.000 | 0.020 | Pseudomonas |
| | ASV11 | 0.001 | 1.732 | 2.299 | Raoultella |
| | ASV110 | 0.038 | 0.000 | 0.006 | Cloacibacterium |
| | ASV117 | 0.038 | 0.000 | 0.016 | Burkholderia |
| | ASV15 | 0.032 | 0.066 | 0.130 | Staphylococcus |

| | | | | | |
|---|---|---|---|---|---|
| | ASV25 | 0.017 | 0.228 | 0.485 | Unclassified Enterobacteriaceae |
| | ASV28 | 0.007 | 0.192 | 0.355 | Kosakonia |
| | ASV36 | 0.007 | 0.014 | 0.353 | Carnobacterium |
| | ASV40 | 0.039 | 0.179 | 0.064 | Pseudomonas |
| | ASV43 | 0.043 | 0.007 | 0.169 | Pantoea |
| | ASV45 | 0.001 | 0.021 | 0.071 | Dermacoccus |
| | ASV48 | 0.039 | 0.033 | 0.047 | Comamonas |
| | ASV73 | 0.002 | 0.004 | 0.040 | Pseudomonas |
| | ASV77 | 0.038 | 0.003 | 0.029 | Micrococcus |
| | ASV8 | 0.005 | 3.051 | 0.943 | Acinetobacter |
| | ASV88 | 0.039 | 0.001 | 0.023 | Paraburkholderia |
| | ASV91 | 0.048 | 0.007 | 0.004 | Morganella |
| | ASV95 | 0.039 | 0.001 | 0.014 | Gordonia |
| Rose Apple | ASV11 | 0.000 | 1.732 | 2.299 | Raoultella |
| | ASV111 | 0.012 | 0.001 | 0.014 | Dechloromonas |
| | ASV13 | 0.012 | 0.044 | 0.059 | Limosilactobacillus |
| | ASV132 | 0.029 | 0.001 | 0.012 | Comamonas |
| | ASV146 | 0.029 | 0.001 | 0.005 | Providencia |
| | ASV15 | 0.031 | 0.066 | 0.130 | Staphylococcus |
| | ASV22 | 0.029 | 0.592 | 0.342 | Lactococcus |
| | ASV3 | 0.001 | 12.420 | 12.107 | Morganella |
| | ASV31 | 0.002 | 0.094 | 0.805 | Klebsiella |
| | ASV36 | 0.002 | 0.014 | 0.353 | Carnobacterium |
| | ASV4 | 0.004 | 1.827 | 10.242 | Providencia |
| | ASV40 | 0.040 | 0.179 | 0.064 | Pseudomonas |
| | ASV43 | 0.012 | 0.007 | 0.169 | Pantoea |
| | ASV44 | 0.029 | 0.048 | 0.018 | Stenotrophomonas |
| | ASV52 | 0.029 | 0.020 | 0.043 | Dermacoccus |
| | ASV6 | 0.000 | 6.190 | 3.537 | Providencia |
| | ASV63 | 0.005 | 0.005 | 0.042 | Pseudomonas |
| | ASV69 | 0.046 | 0.013 | 0.010 | Unclassified Enterobacteriaceae |
| | ASV7 | 0.029 | 4.264 | 1.222 | Unclassified Enterobacteriaceae |
| | ASV73 | 0.002 | 0.004 | 0.040 | Pseudomonas |
| | ASV77 | 0.028 | 0.003 | 0.029 | Micrococcus |
| | ASV88 | 0.029 | 0.001 | 0.023 | Paraburkholderia |
| | ASV9 | 0.031 | 2.187 | 2.605 | Enterococcus |
| Tropical Almond | ASV15 | 0.037 | 0.066 | 0.130 | Staphylococcus |

| | | | | |
|---|---|---|---|---|
| ASV163 | 0.035 | 0.002 | 0.000 | Unclassified Enterobacteriaceae |
| ASV31 | 0.000 | 0.094 | 0.805 | Klebsiella |
| ASV36 | 0.017 | 0.014 | 0.353 | Carnobacterium |
| ASV43 | 0.002 | 0.007 | 0.169 | Pantoea |
| ASV77 | 0.047 | 0.003 | 0.029 | Micrococcus |
| ASV8 | 0.001 | 3.051 | 0.943 | Acinetobacter |

Supplemental Table 3: Wilcoxon rank sum tests comparing ASVs of ovipositor tissues across different fruits. Table shows statistically significant ASVs. Numbers show relative abundance (%).

| | | Relative Abundance (%) | | | | | |
|---|---|---|---|---|---|---|---|
| ASV | P-value | Tropical Almond | Guava | Mango | Papaya | Rose Apple | Taxon |
| ASV127 | 0.001 | 0 | 0 | 0.145 | 0 | 0 | Pseudoxanthomonas |
| ASV150 | 0.006 | 0 | 0 | 0.114 | 0 | 0 | Frateuria |
| ASV26 | 0.050 | 0 | 0.014 | 0.136 | 0.008 | 0.001 | Listeria |
| ASV3 | 0.050 | 22.791 | 0.299 | 36.254 | 7.838 | 1.094 | Morganella |
| ASV4 | 0.033 | 2.857 | 0.042 | 0.086 | 1.572 | 42.756 | Providencia |
| ASV8 | 0.050 | 1.821 | 2.188 | 0.275 | 0.193 | 0.285 | Acinetobacter |

Supplemental Table 4: Wilcoxon rank sum tests comparing ASVs of gut tissues across different fruits. Table shows statistically significant ASVs. Numbers show relative abundance (%).

| | | Relative Abundance (%) | | | | | |
|---|---|---|---|---|---|---|---|
| ASV | P-value | Tropical Almond | Guava | Mango | Papaya | Rose Apple | Taxon |
| ASV10 | 0.011 | 0.752 | 1.4 | 2.959 | 0.016 | 8.768 | Serratia |
| ASV11 | 0.011 | 6.32 | 1.879 | 0.655 | 0 | 0.006 | Raoultella |
| ASV14 | 0.002 | 0.005 | 0 | 0.272 | 0.089 | 0 | Unclassified Bacillaceae 1 |
| ASV15 | 0.014 | 0.005 | 0.004 | 0.269 | 0.066 | 0.001 | Staphylococcus |
| ASV163 | 0.014 | 0.013 | 0 | 0 | 0 | 0 | Unclassified Enterobacteriaceae |
| ASV181 | 0.004 | 0 | 0 | 0.031 | 0 | 0 | Unclassified Saccharibacteria_genera_incertae_sedis |
| ASV21 | 0.001 | 0 | 1.846 | 0 | 0 | 0 | Acinetobacter |
| ASV22 | 0.049 | 1.145 | 0 | 1.738 | 0.227 | 0 | Lactococcus |
| ASV24 | 0.014 | 0 | 0 | 4.4 | 0 | 0 | Flavobacterium |
| ASV26 | 0.03 | 0 | 0.003 | 0.178 | 0.034 | 0 | Listeria |
| ASV28 | 0.011 | 0 | 0.897 | 0 | 0 | 0 | Kosakonia |
| ASV29 | 0.002 | 0 | 0.971 | 0 | 0 | 0 | Klebsiella |
| ASV3 | 0.002 | 15.141 | 0.025 | 18.238 | 31.791 | 0.006 | Morganella |
| ASV32 | 0.006 | 0.234 | 0.022 | 0.536 | 0.377 | 0 | Fructobacillus |
| ASV33 | 0.02 | 0 | 0.474 | 0.538 | 0 | 0.047 | Pseudomonas |
| ASV34 | 0.049 | 0 | 0.15 | 1.327 | 0.002 | 0.072 | Sphingobacterium |
| ASV35 | 0.014 | 0 | 0 | 0 | 0.725 | 0 | Unclassified Enterobacteriaceae |
| ASV4 | 0.049 | 1.797 | 0 | 0.027 | 3.797 | 3.532 | Providencia |
| ASV44 | 0.004 | 0 | 0.19 | 0 | 0 | 0.033 | Stenotrophomonas |
| ASV48 | 0.001 | 0 | 0.156 | 0 | 0 | 0 | Comamonas |
| ASV49 | 0.011 | 0 | 0.001 | 0.664 | 0 | 0 | Chryseobacterium |
| ASV51 | 0.011 | 0 | 0 | 0.52 | 0.002 | 0 | Pedobacter |
| ASV53 | 0.014 | 0 | 0 | 0.469 | 0 | 0 | Variovorax |
| ASV55 | 0.014 | 0 | 0 | 0.35 | 0 | 0 | Delftia |
| ASV58 | 0.014 | 0.011 | 0.086 | 0 | 0 | 0 | Pedobacter |
| ASV6 | 0.014 | 2.892 | 0.142 | 19.77 | 9.659 | 0.014 | Providencia |
| ASV62 | 0.014 | 0 | 0 | 0.325 | 0 | 0 | Caulobacter |
| ASV65 | 0.011 | 0 | 0.065 | 0 | 0 | 0 | Stenotrophomonas |
| ASV7 | 0.002 | 22.019 | 0.018 | 0.347 | 0 | 0 | Unclassified Enterobacteriaceae |
| ASV70 | 0.024 | 0 | 0.072 | 0.033 | 0 | 0.001 | Agrobacterium |
| ASV8 | 0.001 | 0.047 | 9.303 | 0 | 0.002 | 4.894 | Acinetobacter |
| ASV85 | 0.014 | 0 | 0 | 0.114 | 0 | 0 | Flavitalea |
