# Supplementary material for: Impact of Larval Food Source on the Stability of the Bactrocera dorsalis Microbiome
Source: Microb Ecol. 2024 Feb 26;87(1):46. doi: 10.1007/s00248-024-02352-9 (PMC10896919; doi:10.1007/s00248-024-02352-9)
Supplement: Supplementary file 1 — Supplementary file1 (PDF 201 KB) [file 248_2024_2352_MOESM1_ESM.pdf]

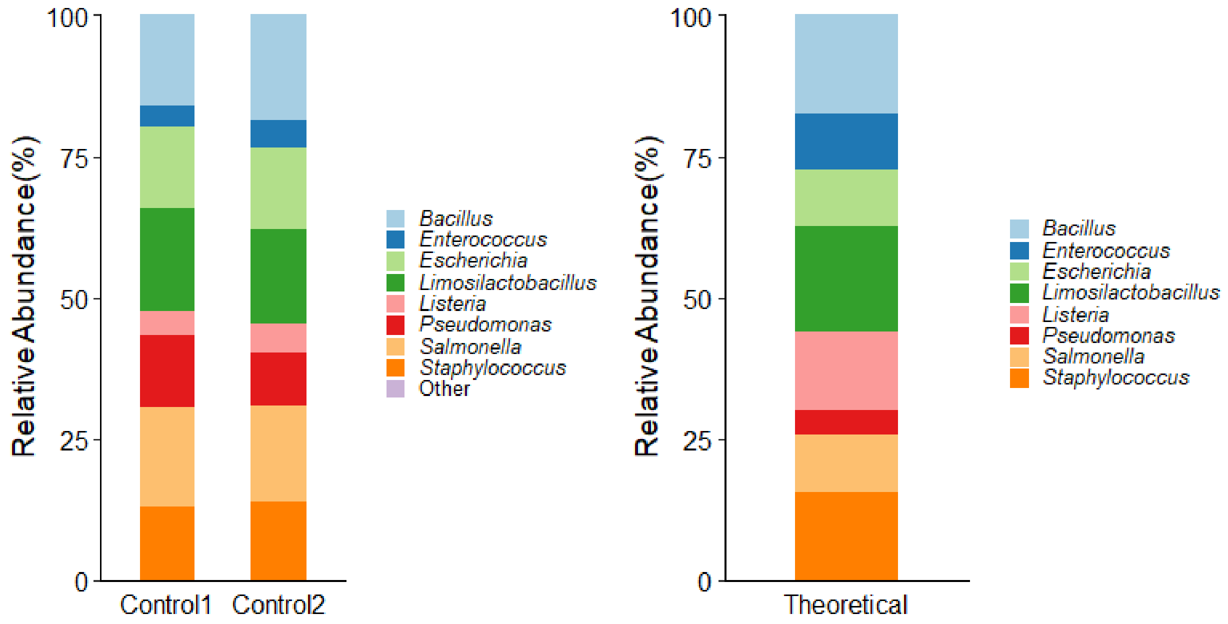

Supplementary Figure 1: Performance of positive microbial extraction control compared to theoretical makeup. 8 ASVs comprised > 99% of the sequences.

Supplementary Table 1: Pairwise PERMANOVA comparisons comparing tissue types across different fruits.

A. Bray-Curtis Comparisons:

	Guava Ovipositor	Mango Gut	Mango Ovipositor	Papaya Gut	Papaya Ovipositor	Rose Apple Gut	Rose Apple Ovipositor	Tropical Almond Gut	Tropical Almond Ovipositor
Guava Gut	0.339	**0.008**	**0.009**	**0.021**	0.193	0.334	**0.009**	**0.008**	0.068
Guava Ovipositor		**0.011**	**0.008**	**0.019**	0.193	0.193	**0.008**	**0.014**	0.068
Mango Gut			0.275	0.232	**0.050**	**0.035**	**0.008**	**0.050**	0.099
Mango Ovipositor				0.193	**0.027**	**0.035**	**0.008**	0.078	0.256
Papaya Gut					0.152	**0.049**	**0.014**	**0.041**	0.367
Papaya Ovipositor						0.193	**0.009**	**0.011**	0.225
Rose Apple Gut							**0.014**	**0.014**	0.080
Rose Apple Ovipositor								**0.009**	**0.020**
Tropical Almond Gut									0.193

B. Jaccard Comparisons

	Guava Ovipositor	Mango Gut	Mango Ovipositor	Papaya Gut	Papaya Ovipositor	Rose Apple Gut	Rose Apple Ovipositor	Tropical Almond Gut	Tropical Almond Ovipositor
Guava Gut	**0.002**	**0.001**	**0.001**	**0.001**	**0.001**	**0.001**	**0.001**	**0.001**	**0.001**
Guava Ovipositor		**0.001**	**0.001**	**0.001**	**0.012**	**0.001**	**0.001**	**0.001**	**0.001**
Mango Gut			**0.001**	**0.001**	**0.001**	**0.001**	**0.001**	**0.001**	**0.001**
Mango Ovipositor				**0.001**	**0.001**	**0.002**	**0.001**	**0.001**	**0.001**
Papaya Gut					**0.001**	**0.001**	**0.001**	**0.001**	**0.001**
Papaya Ovipositor						**0.001**	**0.05**	**0.001**	**0.02**
Rose Apple Gut							**0.001**	**0.001**	**0.001**
Rose Apple Ovipositor								**0.001**	0.307
Tropical Almond Gut									**0.013**

Supplementary Table 2: Wilcoxon rank sum tests comparing ASVs of different host tissues across different fruits. Table shows statistically significant ASVs. Numbers show relative abundance (%).

	ASV	P-Value	Gut Rel Abund	Ovipositor Rel Abund	Taxon
Guava	ASV107	0.029	0.001	0.017	Lactococcus
	ASV11	0.012	1.732	2.299	Raoultella
	ASV117	0.029	0.000	0.016	Burkholderia
	ASV15	0.006	0.066	0.130	Staphylococcus
	ASV29	0.024	0.208	0.033	Klebsiella
	ASV3	0.001	12.420	12.107	Morganella
	ASV31	0.002	0.094	0.805	Klebsiella
	ASV45	0.030	0.021	0.071	Dermacoccus
	ASV6	0.009	6.190	3.537	Providencia
	ASV7	0.010	4.264	1.222	Unclassified Enterobacteriaceae
Mango	ASV10	0.015	2.889	1.188	Serratia
	ASV101	0.017	0.000	0.020	Pseudomonas
	ASV127	0.002	0.000	0.025	Pseudoxanthomonas
	ASV150	0.006	0.000	0.020	Frateuria
	ASV165	0.047	0.000	0.018	Pseudomonas
	ASV166	0.017	0.000	0.013	Novosphingobium
	ASV181	0.016	0.006	0.000	Unclassified Saccharibacteria_genera_incertae_sedis
	ASV24	0.038	0.838	0.000	Flavobacterium
	ASV32	0.007	0.223	0.073	Fructobacillus
	ASV43	0.026	0.007	0.169	Pantoea
	ASV49	0.016	0.127	0.002	Chryseobacterium
	ASV53	0.038	0.089	0.000	Variovorax
	ASV62	0.038	0.062	0.000	Caulobacter
	ASV8	0.002	3.051	0.943	Acinetobacter
	ASV85	0.038	0.022	0.000	Flavitalea
	ASV87	0.038	0.024	0.000	Rhodopseudomonas
Papaya	ASV10	0.004	2.889	1.188	Serratia
	ASV100	0.038	0.000	0.016	Enhydrobacter
	ASV101	0.017	0.000	0.020	Pseudomonas
	ASV11	0.001	1.732	2.299	Raoultella
	ASV110	0.038	0.000	0.006	Cloacibacterium
	ASV117	0.038	0.000	0.016	Burkholderia
	ASV15	0.032	0.066	0.130	Staphylococcus

	ASV25	0.017	0.228	0.485	Unclassified Enterobacteriaceae
	ASV28	0.007	0.192	0.355	Kosakonia
	ASV36	0.007	0.014	0.353	Carnobacterium
	ASV40	0.039	0.179	0.064	Pseudomonas
	ASV43	0.043	0.007	0.169	Pantoea
	ASV45	0.001	0.021	0.071	Dermacoccus
	ASV48	0.039	0.033	0.047	Comamonas
	ASV73	0.002	0.004	0.040	Pseudomonas
	ASV77	0.038	0.003	0.029	Micrococcus
	ASV8	0.005	3.051	0.943	Acinetobacter
	ASV88	0.039	0.001	0.023	Paraburkholderia
	ASV91	0.048	0.007	0.004	Morganella
	ASV95	0.039	0.001	0.014	Gordonia
Rose Apple	ASV11	0.000	1.732	2.299	Raoultella
	ASV111	0.012	0.001	0.014	Dechloromonas
	ASV13	0.012	0.044	0.059	Limosilactobacillus
	ASV132	0.029	0.001	0.012	Comamonas
	ASV146	0.029	0.001	0.005	Providencia
	ASV15	0.031	0.066	0.130	Staphylococcus
	ASV22	0.029	0.592	0.342	Lactococcus
	ASV3	0.001	12.420	12.107	Morganella
	ASV31	0.002	0.094	0.805	Klebsiella
	ASV36	0.002	0.014	0.353	Carnobacterium
	ASV4	0.004	1.827	10.242	Providencia
	ASV40	0.040	0.179	0.064	Pseudomonas
	ASV43	0.012	0.007	0.169	Pantoea
	ASV44	0.029	0.048	0.018	Stenotrophomonas
	ASV52	0.029	0.020	0.043	Dermacoccus
	ASV6	0.000	6.190	3.537	Providencia
	ASV63	0.005	0.005	0.042	Pseudomonas
	ASV69	0.046	0.013	0.010	Unclassified Enterobacteriaceae
	ASV7	0.029	4.264	1.222	Unclassified Enterobacteriaceae
	ASV73	0.002	0.004	0.040	Pseudomonas
	ASV77	0.028	0.003	0.029	Micrococcus
	ASV88	0.029	0.001	0.023	Paraburkholderia
	ASV9	0.031	2.187	2.605	Enterococcus
Tropical Almond	ASV15	0.037	0.066	0.130	Staphylococcus

ASV163	0.035	0.002	0.000	Unclassified Enterobacteriaceae
ASV31	0.000	0.094	0.805	Klebsiella
ASV36	0.017	0.014	0.353	Carnobacterium
ASV43	0.002	0.007	0.169	Pantoea
ASV77	0.047	0.003	0.029	Micrococcus
ASV8	0.001	3.051	0.943	Acinetobacter

Supplemental Table 3: Wilcoxon rank sum tests comparing ASVs of ovipositor tissues across different fruits. Table shows statistically significant ASVs. Numbers show relative abundance (%).

		Relative Abundance (%)					
ASV	P-value	Tropical Almond	Guava	Mango	Papaya	Rose Apple	Taxon
ASV127	0.001	0	0	0.145	0	0	Pseudoxanthomonas
ASV150	0.006	0	0	0.114	0	0	Frateuria
ASV26	0.050	0	0.014	0.136	0.008	0.001	Listeria
ASV3	0.050	22.791	0.299	36.254	7.838	1.094	Morganella
ASV4	0.033	2.857	0.042	0.086	1.572	42.756	Providencia
ASV8	0.050	1.821	2.188	0.275	0.193	0.285	Acinetobacter

Supplemental Table 4: Wilcoxon rank sum tests comparing ASVs of gut tissues across different fruits. Table shows statistically significant ASVs. Numbers show relative abundance (%).

		Relative Abundance (%)					
ASV	P-value	Tropical Almond	Guava	Mango	Papaya	Rose Apple	Taxon
ASV10	0.011	0.752	1.4	2.959	0.016	8.768	Serratia
ASV11	0.011	6.32	1.879	0.655	0	0.006	Raoultella
ASV14	0.002	0.005	0	0.272	0.089	0	Unclassified Bacillaceae 1
ASV15	0.014	0.005	0.004	0.269	0.066	0.001	Staphylococcus
ASV163	0.014	0.013	0	0	0	0	Unclassified Enterobacteriaceae
ASV181	0.004	0	0	0.031	0	0	Unclassified Saccharibacteria_genera_incertae_sedis
ASV21	0.001	0	1.846	0	0	0	Acinetobacter
ASV22	0.049	1.145	0	1.738	0.227	0	Lactococcus
ASV24	0.014	0	0	4.4	0	0	Flavobacterium
ASV26	0.03	0	0.003	0.178	0.034	0	Listeria
ASV28	0.011	0	0.897	0	0	0	Kosakonia
ASV29	0.002	0	0.971	0	0	0	Klebsiella
ASV3	0.002	15.141	0.025	18.238	31.791	0.006	Morganella
ASV32	0.006	0.234	0.022	0.536	0.377	0	Fructobacillus
ASV33	0.02	0	0.474	0.538	0	0.047	Pseudomonas
ASV34	0.049	0	0.15	1.327	0.002	0.072	Sphingobacterium
ASV35	0.014	0	0	0	0.725	0	Unclassified Enterobacteriaceae
ASV4	0.049	1.797	0	0.027	3.797	3.532	Providencia
ASV44	0.004	0	0.19	0	0	0.033	Stenotrophomonas
ASV48	0.001	0	0.156	0	0	0	Comamonas
ASV49	0.011	0	0.001	0.664	0	0	Chryseobacterium
ASV51	0.011	0	0	0.52	0.002	0	Pedobacter
ASV53	0.014	0	0	0.469	0	0	Variovorax
ASV55	0.014	0	0	0.35	0	0	Delftia
ASV58	0.014	0.011	0.086	0	0	0	Pedobacter
ASV6	0.014	2.892	0.142	19.77	9.659	0.014	Providencia
ASV62	0.014	0	0	0.325	0	0	Caulobacter
ASV65	0.011	0	0.065	0	0	0	Stenotrophomonas
ASV7	0.002	22.019	0.018	0.347	0	0	Unclassified Enterobacteriaceae
ASV70	0.024	0	0.072	0.033	0	0.001	Agrobacterium
ASV8	0.001	0.047	9.303	0	0.002	4.894	Acinetobacter
ASV85	0.014	0	0	0.114	0	0	Flavitalea
